# Shifting gears: Id3 enables recruitment of E proteins to new targets during T cell development and differentiation

**DOI:** 10.3389/fimmu.2022.956156

**Published:** 2022-08-02

**Authors:** Michele K. Anderson

**Affiliations:** ^1^ Department of Biological Sciences, Sunnybrook Research Institute, Toronto, ON, Canada; ^2^ Department of Immunology, University of Toronto, Toronto, ON, Canada

**Keywords:** thymus, T-cell development, transcription factor, chromatin, Id proteins, E proteins

## Abstract

Shifting levels of E proteins and Id factors are pivotal in T cell commitment and differentiation, both in the thymus and in the periphery. Id2 and Id3 are two different factors that prevent E proteins from binding to their target gene cis-regulatory sequences and inducing gene expression. Although they use the same mechanism to suppress E protein activity, Id2 and Id3 play very different roles in T cell development and CD4 T cell differentiation. Id2 imposes an irreversible choice in early T cell precursors between innate and adaptive lineages, which can be thought of as a railway switch that directs T cells down one path or another. By contrast, Id3 acts in a transient fashion downstream of extracellular signals such as T cell receptor (TCR) signaling. TCR-dependent Id3 upregulation results in the dislodging of E proteins from their target sites while chromatin remodeling occurs. After the cessation of Id3 expression, E proteins can reassemble in the context of a new genomic landscape and molecular context that allows induction of different E protein target genes. To describe this mode of action, we have developed the “Clutch” model of differentiation. In this model, Id3 upregulation in response to TCR signaling acts as a clutch that stops E protein activity (“clutch in”) long enough to allow shifting of the genomic landscape into a different “gear”, resulting in accessibility to different E protein target genes once Id3 decreases (“clutch out”) and E proteins can form new complexes on the DNA. While TCR signal strength and cytokine signaling play a role in both peripheral and thymic lineage decisions, the remodeling of chromatin and E protein target genes appears to be more heavily influenced by the cytokine milieu in the periphery, whereas the outcome of Id3 activity during T cell development in the thymus appears to depend more on the TCR signal strength. Thus, while the Clutch model applies to both CD4 T cell differentiation and T cell developmental transitions within the thymus, changes in chromatin accessibility are modulated by biased inputs in these different environments. New emerging technologies should enable a better understanding of the molecular events that happen during these transitions, and how they fit into the gene regulatory networks that drive T cell development and differentiation.

## Introduction

Conventional T cells acquire their functional properties in two main phases. The first occurs in the thymus, as T cells transit through successive stages that install the gene expression programs that will run at steady state. The second phase of differentiation occurs in the periphery after exposure to signals that occur during an immune response. These signals activate accessible but latent sub-routines that are kept in check prior to the initiation of the immune response. Both processes depend on the activity of E protein transcription factors and their antagonists, the Id factors. One of the most intriguing aspects of E proteins is their context-dependent use in many different T cell lineages, and the propensity of T cell receptor (TCR) signaling and Id3 activity, in collaboration with other extracellular signals, to create those contexts. While TCR signaling is required for peripheral CD4 T cell differentiation, the specific functional pathways accessed in the periphery are very sensitive to the cytokine milieu. By contrast, the progression of T cell precursors into different pathways in the thymus appears to be driven more by TCR signal strength. In both cases, TCR-dependent upregulation of Id3 is important for allowing changes in changes in chromatin remodeling and gene expression that are needed to restrict E protein activity to the appropriate targets.

## T helper cell differentiation and function

**Figure 1:**
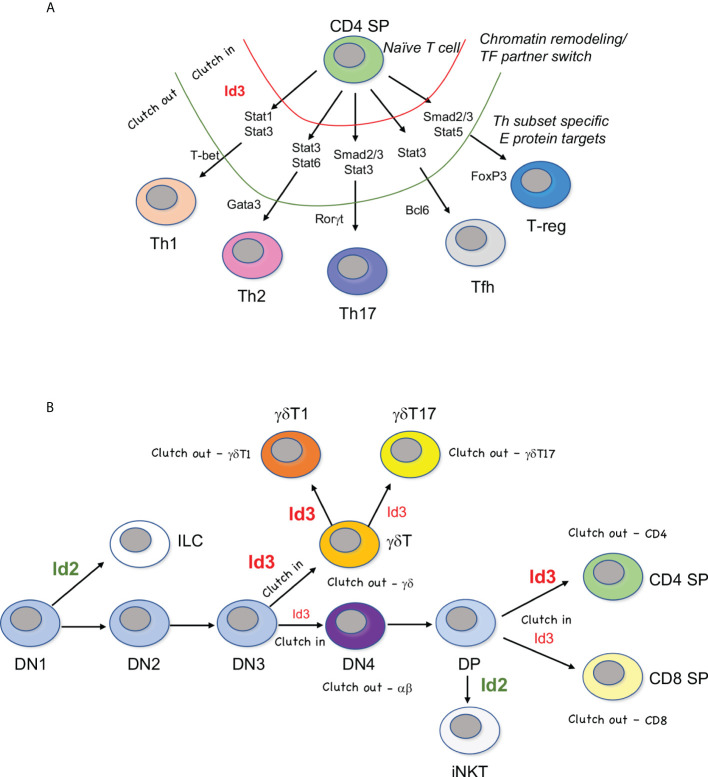
“Clutch” model of Id3-E protein mediated fate choice. **(A)** E proteins regulate a core CD4 T cell program in naïve T cells. Upregulation of Id3 causes a transient inhibition of E protein activity (red, clutch in), during which time initiating transcription factors provide access to Th subset-specific genes. Once remodeling is finished, Id3 activity ceases and E protein activity resumes (green, clutch out). E proteins can then induce master regulators and effector genes specific to each Th lineage. **(B)** Waves of graded Id3 (red) induction in response to TCR signaling pauses a subset E protein activity (clutch in) followed by reassembly of E proteins at successive stages of T cell development (clutch out). Levels of Id3 dictate lineage choice, but E proteins are often engaged in both choices downstream of lineage commitment. Id2 (green) is responsible for differentiation away from the adaptive T cell lineage and into the innate lymphoid cell lineage (ILC) or invariant natural killer T (iNKT) cell lineage. DN=double negative CD4-CD8-, DP=double positive CD4+CD8+, γδT1 = IFNγ-producing γδ T cells, γδT17 = IL-17 producing γδ T cells.

Conventional CD4 T cells emerge from the thymus as “naïve” cells ready for activation. The functional T helper cell differentiation pathways they take upon antigen encounter depends on the types of inflammatory molecules produced during the innate immune response (1) (**Figure 1A**). Each T helper cell subset is dependent on a specific “master regulator” transcription factor that directly induces the effector genes of each program (2). The Th17 lineage, characterized by secretion of IL-17A, IL-17F, and IL-22, is triggered by the innate response to bacteria and fungi. RORγt (*Rorc*) is the Th17 master regulator. Viruses and other intracellular pathogens induce differentiation into the T-bet (*Tbx21*) dependent Th1 pathway, leading to IL-2, TFNα, and IFNγ production. Helminth infection induces the Th2 fate, leading to secretion IL-4, IL-5, and IL-13, under the control of GATA3 (3).

Other Th subsets generated in the periphery include Bcl6-driven T-follicular helper cells (Tfh) (4), specialized for B cell help in the germinal center, and induced T-reg cells, which, like thymic-derived T-regs, depend on FoxP3 (5). In addition to playing unique roles in immunity, Th subsets also have pathogenic impacts when dysregulated (6). In general, Th1 and Th17 cells contribute to autoimmune pathology, Th2 cells are largely responsible for allergic reactions, and T-regs inhibit anti-cancer immunity (7, 8). Most Th subsets retain plasticity after activation, and some can transdifferentiate from one type to another (2). Additional Th subsets continue to be identified, including Th22, Th9, Tfh13, and Tr1 cells, suggesting that the networks controlling these effector functions are dynamic, and represent more of a physiological state than a committed fate, rendering them open to manipulation during an immune response (9–11).

## Transcriptional control of Th differentiation

Differentiation of naïve CD4 T cells into the Th subsets is coordinated by several sets of signal-dependent transcription factors (12). Triggering of the αβ TCR and co-stimulatory receptors leads to activation of NFkB, NFAT, and IRF transcription family members, as well as upregulation of AP1 transcription factor family members such as BATF and Jun (13, 14). Cytokine receptor signaling leads to the activation of different sets of transcription factors, most notably members of the STAT and SMAD families (15, 16). BATF, IRF4, and the cytokine-responsive factors recruit chromatin remodeling enzymes that provide access to genes of specific Th subsets, while restricting access to genes of alternative Th subsets (13, 17). After chromatin remodeling, the master regulators are induced, providing the final key needed for functional activation during the immune response.

E proteins and Id proteins are involved in regulation of the naïve CD4 T cell state, and in the differentiation of Th2, Th17, and T-reg cells (18–20). In general, E protein activity is regulated post-translationally by Id proteins, which sequester them in inactive dimers. The requirement for E proteins for Th17 differentiation has been especially well studied. A comprehensive study conducted by the Strober group in 2013 showed that mice carrying a conditional double HEB/E2A deletion on a CD4-Cre background had a profound defect in Th17 development *in vitro*, and compromised immune function *in vivo*, using both autoimmunity and infection models (21). This study also showed that HEB and E2A can directly bind and activate the *Rorc* locus, but only in the context of Th17 cells, not in naïve CD4 T cells. Studies of Id3-deficient mice suggest that E proteins restrain the Th2 and Tfh lineages and promote the Th9 lineage, whereas Th1 cells appear to require Id proteins and to be E protein independent (21–24). Interestingly, T-regs require both Id3 and E2A in a sequential manner. TGFβ induces transient expression of Id3, which is needed to prevent repression of the FoxP3 promoter (25). This repression is not mediated directly by E proteins, but rather results from E protein-mediated upregulation of GATA3. Subsequently, E2A activity is required to directly activate the FoxP3 promoter. However, if E2A levels are too high, FoxP3 expression becomes unstable in T-regs, emphasizing the importance of transcription factors levels in maintaining stable outcomes (26).

## The Clutch model of E protein/Id3 activity in T cell transitional states

The theme of transient Id3 expression followed by shifting E protein target gene activation suggests what we term a “Clutch” model of Th differentiation (**Figure 1**). In this model, Id3-mediated pausing of E protein activity would act like the clutch of a car, withholding access to the engine (E protein activity) until the appropriate gear (chromatin context) is engaged, and then allowing the engine to move the car (Th differentiation) forward in a controlled fashion (**Figure 1A**). E proteins bind to many effector genes in Th subsets. Therefore, it is likely that restriction of E protein binding to the “right” set of mediators within each lineage is essential for linking environmental input to functional output in Th subsets. This is clearly a strong paradigm for peripheral T cell differentiation (27). The Clutch model also applies to T cell development in the thymus, but with a twist, as described below (**Figure 1B**). Moreover, the role of Id2 in thymic T cell development exhibits stark differences from Id3 during thymic development and does not conform to the Clutch model.

## Id2 regulates the innate/adaptive fate choice in early T cell precursors

The earliest T cell progenitors (ETPs) to enter the thymus are not yet committed to the T-cell lineage and have alternative fates available to them depending on their access to microenvironmental signals. One of the key molecular switches that must be flipped to gain access to the T cell pathway is to increase E protein activity. This occurs in at least two different ways. The first is upregulation of E proteins at the mRNA level, and the second is the downregulation of Id2 (28). Id2 is a critical mediator of the innate/adaptive lineage split (18, 29). ETPs express “legacy genes”, thus termed because they are expressed in hematopoietic stem cells (30). ETP legacy genes include Id2, the Ets protein PU.1, and the Class II bHLH factor SCL. All three of these factors can act in opposition to T-lineage commitment: PU.1 drives expression of myeloid and B cell genes (31), SCL can re-direct E proteins to stem cell gene loci and away from T cell gene loci (32), and Id2 interferes with E protein activity. E protein activity is essential for the expression of Rag recombinase genes, which are necessary for the generation of TCRs and thus T cells (33). Unlike Id3, Id2 does not appear to be under the influence of transient signals during thymocyte development but rather is subject to degradation in a cell cycle-dependent manner (34, 35). Downregulation of PU.1 and upregulation of Bcl11b in early T cell development results in the cessation of Id2 mRNA expression, which allows upregulation of T-lineage E protein target genes (36, 37). Conversely, Id2 expression is maintained in mature innate cells including ILCs, NK cells, and myeloid cells, and appears to support the maintenance of lineage fidelity.

## Notch signaling shifts the E protein-Id2 balance to allow T cell development

**Figure 2:**
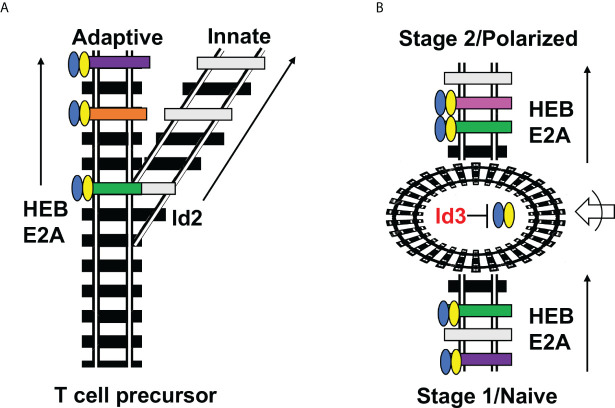
Railroad analogy of Id2 versus Id3 outcomes. **(A)** Id2 acts as a switch that diverts T cell precursors away from the adaptive fate and towards the innate fate by permanently silencing E protein activity and E protein target gene expression. **(B)** Id3 serves as a way station (roundhouse) that allows changes in accessibility of E protein target genes while E proteins are inactive, followed by E protein engagement with different E protein target genes at the next stage of development. This occurs in both CD4 T cell differentiation (Naïve/Polarized) and multiple stages of thymic differentiation (Stage 1/Stage 2). Blue-Yellow ovals = HEB/E2A. Black railroad ties = completely inaccessible genes; gray railroad ties = accessible genes lacking the proper combination of transcription factors for induction; colored railroad ties = E protein target genes bound by HEB/E2A and undergoing active transcription. Roundabout = Id3-mediated pause in E protein activity during which changes in accessibility of E protein target sites occurs. Arrow with half circle = extracellular signaling inputs that direct which genes undergo changes in chromatin accessibility.

As ETPs enter the thymus, they are exposed to Delta-like (Dll) ligands of Notch receptors, resulting in strong Notch signaling. Notch signaling is indispensable for T cell specification and lineage commitment, acting upstream of an elegant cascade of transcription factors that inhibits alternative fates and induces T-cell genes (38). While Notch regulates a wide swath of important target genes, one of the most important roles plays in T-lineage commitment is by shifting the balance between Id and E protein activity in ETPs, in three complimentary ways. First, Notch redirects PU.1 away from Id2 and towards more T-lineage friendly genes (39). Secondly, Notch upregulates the E protein HEBAlt, increasing the overall E protein availability (40). Thirdly, Notch directly upregulates Bcl11b, which downregulates Id2 at the transcriptional level (37). This delivers a one-two-three punch that directs cells permanently away from Id2-dependent ILCs and into the T-cell lineage. Thus, Id2 does not acts as a way station for changing gene availability to E proteins, but instead is more akin to a railroad switch that directs cells down one pathway or another (**Figure 2**).

## TCR signal strength determines lineage outcomes during the intrathymic T cell lineage choices

Once cells have been switched onto the T-lineage track, they progress towards the first “checkpoint” of T cell development. There are two main checkpoints that occur during T cell development, so called because they serve as testing of the cells for functional TCR rearrangement and function (**Figure 1B**). During the first checkpoint, the TCRβ chain pairs with the pre-Tα chain to form a pre-TCR. The only requirement for the pre-TCR to allow “passage” through the checkpoint is for it to complex with CD3 chains and translocate to the cell membrane long enough to invoke a weak set of signaling cascades (41). Alternatively, the cell can rearrange and express a TCR composed of TCRγ and TCRδ chains. In this situation, the γδ TCR/CD3 complex is stably expressed on the surface, transmitting a stronger signal than that transduced by the pre-TCR, which directs cells away from the αβ T cell fate and into the γδ T cell fate (42, 43). After commitment to the αβ T cell lineage, cells expressing αβ TCRs are subjected to second “checkpoint” which vets these TCRs for their ability to bind to MHC/peptide and assesses the affinity of the interaction. As with the first checkpoint, this signal also serves as a lineage branchpoint, with cells experiencing lower and briefer TCR signaling adopting the CD8 fate, and cells experiencing longer and stronger TCR signaling progressing into the CD4 T cell lineage (44). This paradigm also applies to committed γδ T cells that progress along the IFNγ-producing γδT1 fate or the γδT17 fate (45) (**Figure 1B**). Engagement of strong γδ TCR ligands in conjunction with co-stimulatory molecules results in strong TCR signaling and the γδT1 developmental outcome, whereas a less strong TCR signal leads to the γδT17 fate (46–48). All these lineage choices are intimately associated with the balance between Id3 and E proteins (49, 50).

## Translation of TCR signal strength into Id3 activity modulates E protein target gene accessibility

As in peripheral CD4 T cells, TCR signaling in early precursors leads to upregulation of Id3, and a pause in E protein activity allows chromatin remodeling and shifting of E protein target availability. There may also be a role for TCR signal strength during T helper cell differentiation, particularly in combination with cytokine signaling (51). However, there is a clear hierarchy of TCR signal strength that is induced at each checkpoint in thymic T cell development (52). During T cell development, TCR signaling may shift the balance between Id3 and E proteins to different degrees, allowing retention of E protein occupancy on some sites but not others. E2A and HEB are direct regulators of most of the genes needed for assembly of the TCR genes and formation of the pre-TCR (53, 54). Id3 is also induced in response to αβTCR signaling at the DP stage, and is necessary to overcome the gatekeeper function of E proteins at the DP to SP transition (55, 56). However, past this checkpoint, E proteins are required for the generation of CD4 SP cells (57). E proteins also regulate genes in γδ-T committed cells that dictate functional programming, including *Tcf7* (58). An elegant study by Hosoya and colleagues shed considerable light on the chromatin remodeling events that occur during αβ T cell development using ATAC-seq, which detects open chromatin and predicts the presence of transcriptional complexes (59). This study showed that the loci for both γδ-lineage and αβ-lineage genes were accessible in DN thymocytes. However, as cells transitioned from the DN to the DP stage and then to the CD4 and CD8 stages, cis-regulatory elements with predicted binding by the key γδ-lineage factor Sox13 showed a dramatic loss of accessibility. Likewise, predicted HEB sites shifted in accessibility according to the stage of αβ T cell development, consistent with Id3-facilitated chromatin remodeling at these transitions. This is doubtless just the beginning of this new phase of our journey towards a deeper understanding of T cell developmental transitions, and it will be exciting to learn how E protein genomic site occupancy changes after they are dislodged and then reassembled on different loci at progressive stages of T cell development and differentiation.

## Limitations of the Clutch model of Id3-facilitated shifts in E protein targets

Like E proteins, Id3 is used widely in different contexts outside of T cell development (60, 61). Clearly, the Clutch model does not apply in all situations, but rather appears to be restricted to certain types of cells and developmental transitions. Moreover, an examination of E2A occupancy at the DN3 to DN4 transition revealed both overlapping and unique sites of E2A occupancy in both subsets, indicating that E2A was only dislodged from a subset of sites during the transition, while others were maintained (19). Release of E proteins from specific sites likely depends on both the Id3/E protein ratio and the availability of E protein binding partners. For instance, the downregulation of Notch1 in response to pre-TCR signaling would be predicted to increase the disengagement of E proteins from sites that require both Notch factors and E proteins, but not from other sites that maintain the core T-lineage program. Importantly, E proteins themselves are important mediators of chromatin remodeling, interacting directly with both positive and negative regulators of chromatin configuration such as p300, CHD4, LSD1, and PRC2 (62–66). It is important to note that chromatin remodeling in this context does not indicate simply a shift between “open” and “closed” configurations, but also includes the transition from “poised” to “active” states (67). This may be mediated in part by fresh access to new binding partners that become available after the transition. Furthermore, the plasticity of CD4 T cell subsets suggests that lineage-specifying E protein sites remain accessible during and after CD4 T cell differentiation (68). A comprehensive understanding of global E protein occupancy changes that occur during these processes awaits further studies. Likewise, the relative contributions of E2A versus HEB to these processes are not well understood.

## Discussion

While it is well understood that Id proteins inhibit E protein activity and interfere with the expression of E protein target genes, much less is known about how E protein targets shift during the developmental transitions that occur during Id3 expression, and the molecular events that underpin them. Here, the Clutch model is presented as a conceptual scaffold that will provoke questions and undergo modifications and stratification as new data is obtained revealing E protein chromatin occupancy before, during, and after T cell stages transitions, and identifying stage-specific E protein partners. Due to technical limitations, earlier studies largely relied on *in vitro* models of T cell development or differentiation such as OP9-DL co-culture derived T cell precursors or *in vitro* polarization of naïve peripheral T cells (69, 70). While these studies have provided a wealth of information into the global events that orchestrate T cell development, they cannot completely replicate the complex thymic niches that shift over time as cells migrate through different niches in the thymus, nor can they fully provide the complex medley of signals that transpire during a coordinated immune response. The advent of single cell RNA-seq, and multiomic approaches such as scRNA-seq/ATAC-seq and CITE-seq that allows that require fewer input cells are now providing unprecedented access to *ex vivo* precursors and products that arise during T cell development. Moreover, computational methods such as pseudotime modeling and RNA velocity are further advancing our understanding of transient states of development (71). Importantly, there is a fourth dimension that is rarely considered in these snapshot approaches: time. Single cell live imaging has revealed that Id3 transcription is “bursty”, occurring in only a small number of cells within a population at any one time, in the B cell lineage (72). It remains to be seen whether this is true in T cell precursors, and whether TCR signaling can synchronize cells into uniformly high Id3 expressers. Alternatively, burstiness may contribute to the gradation of Id3 that mediates intrathymic T cell fate choices. By contrast, Id2 acts as a permanent switch into the innate lineage choice. This distinction highlighting the unique nature of Id3 in regulating fate choices by facilitating E protein target changes as T cells journey through development in the thymus or differentiate in the periphery during an immune response.

## Author contributions

The author confirms being the sole contributor of this work and has approved it for publication.

## Funding

The ideas presented in this work were made possible by support from NIH (1P01AI102853-06), CIHR (201610PJT) and NSERC (RGPIN 05333-14).

## Acknowledgments

The ideas presented in this Mini Review have been strongly influenced by my conversations with colleagues too numerous to present here, and I would like to apologize to any that were not cited due to space limitations. I am particularly indebted to Juan Carlos Zúñiga-Pflücker, David Wiest, Yuan Zhuang, and Cornelis Murre for thoughtful and lively discussions and for their ongoing contributions to the field.

## Conflict of interest

The author declares that the research was conducted in the absence of any commercial or financial relationships that could be construed as a potential conflict of interest.

## Publisher’s note

All claims expressed in this article are solely those of the authors and do not necessarily represent those of their affiliated organizations, or those of the publisher, the editors and the reviewers. Any product that may be evaluated in this article, or claim that may be made by its manufacturer, is not guaranteed or endorsed by the publisher.
